# DNA nanostructure‐programmed intermembrane spacing to modulate T‐cell immunity

**DOI:** 10.1002/ctm2.1379

**Published:** 2023-08-20

**Authors:** Yulin Du, Liping Qiu, Weihong Tan

**Affiliations:** ^1^ The Key Laboratory of Zhejiang Province for Aptamers and Theranostics Zhejiang Cancer Hospital Hangzhou Institute of Medicine (HIM) Chinese Academy of Sciences Hangzhou Zhejiang China; ^2^ Molecular Science and Biomedicine Laboratory (MBL) State Key Laboratory of Chemo/Biosensing and Chemometrics College of Chemistry and Chemical Engineering College of Biology Aptamer Engineering Center of Hunan Province Hunan University Changsha Hunan China; ^3^ Institute of Molecular Medicine (IMM) Renji Hospital Shanghai Jiao Tong University School of Medicine and College of Chemistry and Chemical Engineering Shanghai Jiao Tong University Shanghai China

## 1 EFFECT OF INTERMEMBRANE SPACING ON TCR SIGNALLING

T‐cell‐mediated adaptive immunity plays a pivotal role in combating numerous diseases. This process is initiated when the T‐cell receptor (TCR) binds to antigenic peptides presented by the major histocompatibility complex (pMHC) on the surface of antigen‐presenting cells (APCs).^1^ Upon TCR–pMHC‐specific binding, a close‐contact zone rapidly forms at the interface between the APC and T cell, with an axial distance of approximately 13 nm.^2^ This close‐contact zone facilitates the phosphorylation of immunoreceptor tyrosine‐based activation motifs (ITAMs) in the cytoplasmic domains of the TCR complex by lymphocyte‐specific protein tyrosine kinase (Lck), triggering a cascade of signalling pathways that lead to T‐cell activation. Despite its crucial role in T‐cell immunity, there is still controversy regarding the molecular mechanism of how TCR–pMHC binding initiates intracellular ITAM phosphorylation.^3^

Previous studies have examined the spatial impact of the close‐contact zone on TCR triggering process by genetically manipulating the length of pMHC ligand or other membrane proteins.^4^, ^5^ Whereas, these techniques involve modifications to protein structure and expression, and show difficulty to accurately assess the linear correlation between intermembrane distance and TCR signalling strength. While artificially engineered nanointerfaces can provide measurable systems, they have limitations in replicating real APCs and capturing the full complexity of this biological process, as they lack many intrinsic membrane‐associated molecules and adaptive morphologies.^6^ Additionally, the existing strategies have mainly focused on extending intermembrane spacing, with minimal exploration on the effects of compressing the intermembrane distance. This gap in research leaves room for further investigation into the underlying mechanisms.

## 2 PROGRAMMABLE DNA NANOJUNCTIONS FOR MODULATING INTERMEMBRANE SPACING WITH NANOMETER PRECISION

**FIGURE 1:**
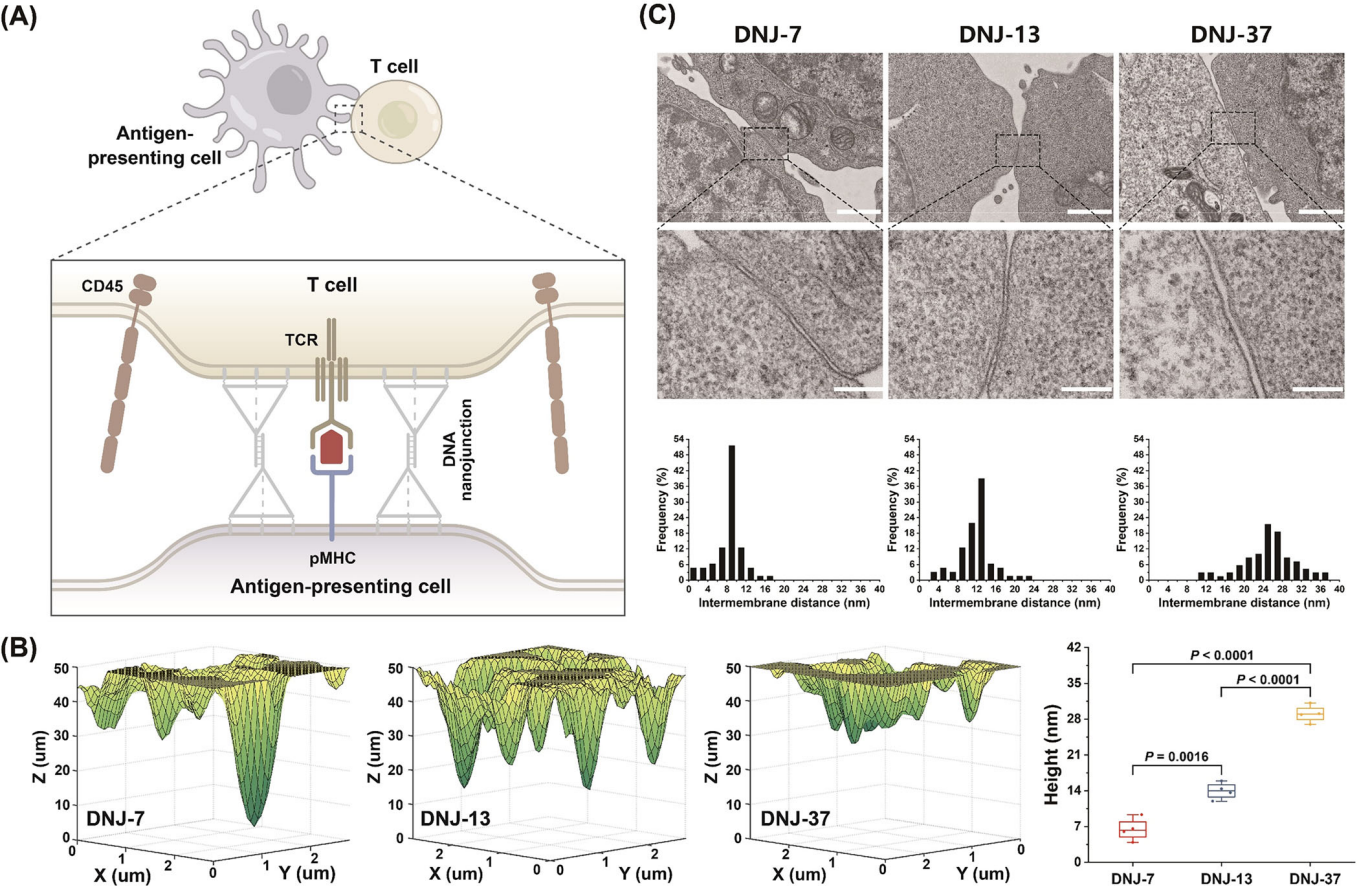
Characterisation of DNA nanojunctions (DNJs) for manipulating the intermembrane spacing. (A) Schematic of the DNJ‐based precise manipulation of the close‐contact zone for studying T‐cell receptor (TCR) signalling. (B) Reconstruction of the three‐dimensional morphology of the close‐contact zone mediated by different DNJ groups, and their statistical analysis of the intermembrane distance, respectively. (C) Transmission electron microscopy (TEM) images of DNJ‐mediated contact between CCRF‐CEM cells (top; scale bar: 1 μm) and the corresponding membrane constraint (dotted frame region) with higher magnification (bottom; scale bar: 200 nm). The intermembrane distances are measured based on the TEM images and grouped in 2‐nm intervals. Reproduced from Ref.^7^ with permission. Copyright 2023 Springer Nature (colour online).

To overcome above limitations, we developed DNA nanojunctions (DNJs) of different sizes, by using cholesterol‐labelled DNA tetrahedrons (TDNs) as the cell membrane‐anchoring scaffolds^7^ (Figure 1A). TDNs were constructed by DNA self‐assembly, displaying an overhang strand at the top vertex and cholesterol tags at the three bottom vertices. Unlike conventional genetic engineering strategies, no protein modification was required in our system. The rapid, effective and stable membrane‐anchoring capability of TDNs was also demonstrated by our previous work.^8^ Meanwhile, the density of TDNs on the cell surface could be easily adjusted by modulating their incubation concentration. To control the intermembrane spacing, we designed three types of DNJs, namely, DNJ‐7, DNJ‐13 and DNJ‐37, corresponding to the theoretical size of 9.6, 13.0 and 26.4 nm, respectively. These DNJs were constructed by hybridising the overhang strands between two TDNs of the same size. Based on total internal reflection fluorescence microscopy (TIRFM) and transmission electron microscopy (TEM) imaging, we showed that these DNJs can precisely tune the intermembrane spacing to the desired values (Figure 1B,C). This capability allowed to extend, maintain and compress the spatial distance of TCR–pMHC‐mediated contact zone in a real system of interaction between APCs and T cells.

## 3 POTENTIAL MOLECULAR MECHANISMS OF TCR SIGNALLING

**FIGURE 2:**
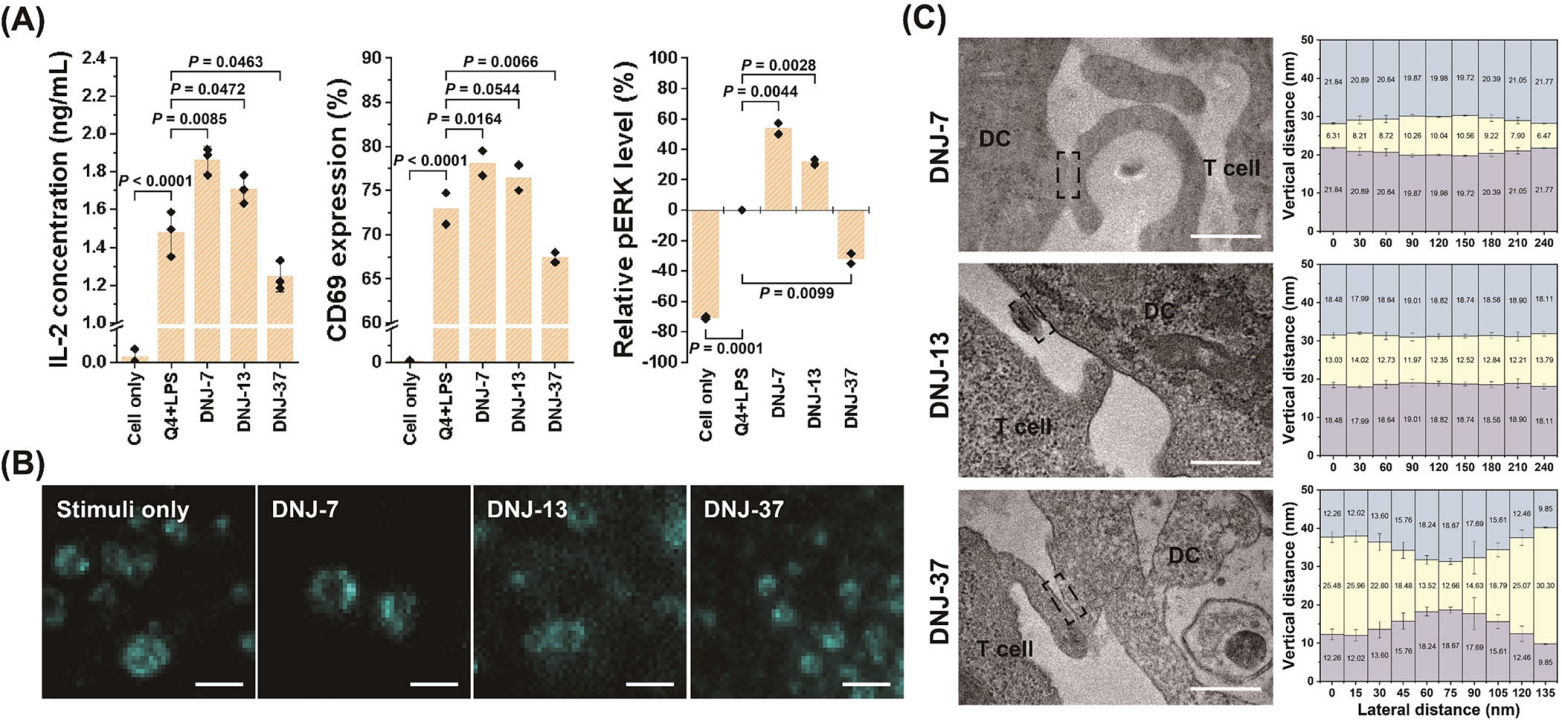
Mechanistic investigation of T‐cell receptor signalling. (A) IL‐2 secretion and CD69 expression of OT‐I T cells after 18‐h incubation with LPS/Q4‐pretreated DCs under the mediation of different DNA nanojunctions (DNJs) (10 nmol/L TDNs). Relative phosphorylation level of ERK1/2 of OT‐I T cells after 15‐min incubation with LPS/Q4‐pretreated DCs under the mediation of different DNJs (10 nmol/L TDNs). (B) TIRFM images of the lateral distribution of recombinant CD45 with the mediation of different DNJs. Scale bar: 2 μm. (C) TEM images of DNJ‐mediated DC–T‐cell contact. The close‐contact zone is marked in the black dotted box, and the axial intermembrane distance was measured at a certain interval along the membrane constraint. Scale bar: 400 nm. Reproduced from Ref.^7^ with permission. Copyright 2023 Springer Nature (colour online).

By using DNJs to manipulate the axial dimension of the cell–cell interface, we observed a consistent trend in T‐cell signalling amplitude: DNJ‐7 > DNJ‐13 > DNJ‐37 (Figure 2A). Additionally, we found that at a critical low surface density, DNJ‐37, which is larger than the size of the TCR–pMHC complex, could weaken T‐cell activation level by increasing the intermembrane distance and reducing CD45 segregation (Figure 2B,C). On the other hand, DNJ‐7, with a smaller size, could compress the intermembrane spacing at the APC–T‐cell interface, resulting in a strict exclusion of CD45 and intensified conformational changes of TCR complex. This compression consequently led to a significant enhancement of T‐cell activation. While DNJ‐13 had a minimal effect on the intermembrane distance, it could effectively stabilise the TCR–pMHC‐mediated cellular interface, leading to prolonged ITAM phosphorylation and stronger T‐cell stimulation compared to DNJ‐free control groups.

## 4 FUTURE OUTLOOK

These findings provide direct evidence for the pivotal role of the axial size of the contact zone in T‐cell triggering. Notably, reducing the intermembrane distance at the APC–T‐cell interface to less than 10 nm could significantly enhance T‐cell activation, thus expanding the opportunities for the study of T‐cell immunity and further applying for the development of novel therapies for immune‐related disorders. Considering that cell–cell interactions through direct contact are prevalent in organisms and the interface dimension is important in modulating various signal transduction processes, this membrane‐anchored DNA nanoplatform, with its high programmability, controllability, ease of operation, and good biocompatibility,^9^, ^10^ offers a promising way for studying these biochemical events. Moreover, it exhibits significant potential in promoting targeted T‐cell responses in the field of immunotherapy.

## CONFLICT OF INTEREST STATEMENT

The authors declare they have no conflicts of interest.

## References

[1] Weiss A , Dan RL . Signal transduction by lymphocyte antigen receptors. Cell. 1994;76(2):263‐274. 10.1016/0092-8674(94)90334-4 8293463

[2] Garcia KC , Degano M , Stanfield RL , et al. An alphabeta T cell receptor structure at 2.5 A and its orientation in the TCR‐MHC complex. Science. 1996;274(5285):209‐219. 10.1126/science.274.5285.209 8824178

[3] Chakraborty AK , Weiss A . Insights into the initiation of TCR signaling. Nat Immunol. 2014;15(9):798‐807. 10.1038/ni.2940 25137454 PMC5226627

[4] Choudhuri K , Wiseman D , Brown MH , Gould KG , Der Merwe PAV . T‐cell receptor triggering is critically dependent on the dimensions of its peptide‐MHC ligand. Nature. 2005;436(7050):578‐582. 10.1038/nature03843 16049493

[5] James JR , Vale RD . Biophysical mechanism of T‐cell receptor triggering in a reconstituted system. Nature. 2012;487(7405):64‐69. 10.1038/nature11220 22763440 PMC3393772

[6] Cai H , Muller JE , Depoil D , et al. Full control of ligand positioning reveals spatial thresholds for T cell receptor triggering. Nat Nanotechnol. 2018;13(7):610‐617. 10.1038/s41565-018-0113-3 29713075 PMC6035778

[7] Du Y , Lyu Y , Lin J , et al. Membrane‐anchored DNA nanojunctions enable closer antigen‐presenting cell‐T‐cell contact in elevated T‐cell receptor triggering. Nat Nanotechnol. 2023;18(7):818‐827. 10.1038/s41565-023-01333-2 36894782

[8] Li J , Xun K , Pei K , et al. Cell‐membrane‐anchored DNA nanoplatform for programming cellular interactions. J Am Chem Soc. 2019;141(45):18013‐18020. 10.1021/jacs.9b04725 31626550

[9] Xia X . DNA nanotechnology for modulating the growth and development of neurons. CCS Chem. 2021;3(9):2381‐2393. 10.31635/ccschem.020.202000456

[10] Zhou Y , Zhuo Y , Peng R , et al. Functional nucleic acid‐based cell imaging and manipulation. Sci China Chem. 2021;64:1817‐1825. 10.1007/s11426-021-1115-3

